# Design and screening of novel molecular compounds targeting lactate dehydrogenase of *Babesia microti*

**DOI:** 10.1186/s13071-024-06623-9

**Published:** 2025-02-23

**Authors:** Wanxin Luo, Long Yu, Shiyu Lu, Yuxin Yu, Yidan Bai, Sen Wang, Dongfang Li, Zhen Han, Yaxin Zheng, Fangjie Li, Junlong Zhao, Lan He

**Affiliations:** 1 National Key Laboratory of Agricultural Microbiology, College of Veterinary Medicine, Huazhong Agricultural University, Wuhan, 430070 Hubei China; 2 Key Laboratory of Preventive Veterinary Medicine in Hubei Province, The Cooperative Innovation Center for Sustainable Pig Production, Wuhan, 430070 Hubei China; 3 Key Laboratory of Development of Veterinary Diagnostic Products, Ministry of Agriculture of the People’s Republic of China, Wuhan, 430070 Hubei China

**Keywords:** *Babesia microti*, Lactate dehydrogenase, Compound, Computer-aided drug design, Surface plasmon resonance

## Abstract

**Background:**

Human babesiosis is caused by several species within the *Babesia* genus, primarily *Babesia microti*, *Babesia duncani*, and *Babesia divergens*, all of which infect human red blood cells (RBCs). Clinically, the disease manifests with symptoms such as fever, anemia, jaundice, and hemoglobinuria, with *B. microti* being the most prevalent of these species. Our previous research has shown that *B. microti* primarily relies on lactate dehydrogenase (LDH)-mediated anaerobic glycolysis, rather than the tricarboxylic acid cycle (TCA cycle), to generate ATP for its intracellular survival. Because LDH is a promising drug target, it can be inhibited by compounds such as gossypol and 3,5-dihydroxy-2-naphthoxylic acid (DHNA). In this study, we conduct a structure-based optimization of DHNA, leading to the development of a novel library of compounds derived from its structure.

**Methods:**

Two compounds were identified and synthesized through molecular docking, on the basis of the crystal structure of *Babesia microti* lactate dehydrogenase (BmLDH). The effects of these compounds were evaluated using several methods, including surface plasmon resonance (SPR) assays, enzyme activity inhibition tests, in vitro growth inhibition assays against *B. microti*, and mammalian cytotoxicity tests.

**Results:**

Compounds target A (TA) (−36.0) and B (TB) (−43.8), both exhibiting low CDOCKER energy values, achieved final purities of 96.6% and 97.5%, respectively. Surface plasmon resonance (SPR) experiments showed that TA and TB had comparable dissociation constant (*K*_D_) values of 11.3 × 10^−6^ M and 13.2 × 10^−6^ M, respectively. However, enzyme activity inhibition assays indicated that TB was more potent, with an half-maximal inhibitory concentration (IC_50_) value of 23.8 μM, compared with TA’s IC_50_ of 71.6 μM. Additionally, TB demonstrated a strong ability to inhibit the in vitro growth of *B. microti*, with an IC_50_ value of 111.7 μM.

**Conclusions:**

In this study, two compounds capable of inhibiting the growth of *B. microti* were obtained. Although both compounds showed moderate inhibitory activity against recombinant BmLDH (rBmLDH) and the growth of *B. microti*, there is potential to enhance their efficacy through further structural modifications, particularly of compound TB.

**Graphical Abstract:**

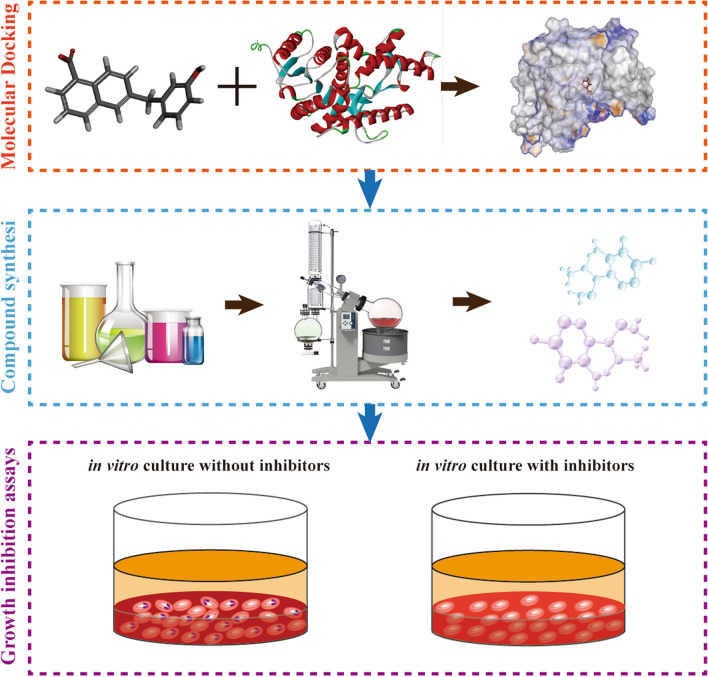

**Supplementary Information:**

The online version contains supplementary material available at 10.1186/s13071-024-06623-9.

## Background

*Babesia microti* is a significant tick-borne pathogen that infects erythrocytes. While it primarily affects small rodents, it can also infect humans [1]. Individuals who are elderly, immunocompromised, or have undergone splenectomy are particularly vulnerable, facing an increased risk of developing multiple organ failure and potentially fatal outcomes [2, 3]. The disease is widespread, with cases reported in various countries, including the USA and China. Notably, the incidence of babesiosis continues to rise globally each year [1, 4]. In 2019, the Centers for Disease Control and Prevention (CDC) reported 2418 human cases of babesiosis in the USA, reflecting an 11% increase from 2018. In China, between 1993 and 2016, 214 cases of babesiosis were documented, with *B. microti* responsible for 46.7% of those cases [5]. Currently, no commercially available vaccine exists for babesiosis, and commonly used treatments such as atovaquone, azithromycin, clindamycin, and quinine are associated with significant side effects [6, 7]. Moreover, drug resistance has emerged as a major challenge. Mutations such as M134I in the atovaquone binding site have been observed following treatment, and the combination of atovaquone with azithromycin can induce mutations such as Y272C in another atovaquone binding site. Prolonged azithromycin use may also lead to mutations (R86C) in the L4 (*RPL4*) gene, which encodes a ribosomal protein subunit in the apicoplast genome, contributing to drug resistance [8–11]. These developments underscore the urgent need for the discovery of novel and targeted therapeutics to effectively combat babesiosis.

Sequencing the genome of *B. microti* revealed that it lacks malate dehydrogenase (MDH), which prevents the completion of the TCA cycle [12]. This strongly suggests that BmLDH-mediated anaerobic glycolysis is the primary pathway for ATP production in *B. microti*. In our previous work, Arg99 in BmLDH was identified as a critical site for drug targeting [13]. Gossypol, a polyphenolic compound derived from the mallow family, has demonstrated strong biological activity against various parasites [14–16]. In vitro studies revealed that gossypol significantly inhibits lactate dehydrogenase (LDH) activity in *B. microti* at a concentration of 592 nM [13]. However, its toxicity limits its potential for clinical application [17]. Despite this limitation, gossypol serves as a valuable lead compound for the development of structural analogues designed to retain efficacy while minimizing toxicity. In 1998, the structure of gossypol was modified to produce a series of derivatives. Among these, 11F (FX11) exhibited the most potent inhibitory effect on *Plasmodium falciparum* LDH, with a *K*^i^ value of 300 nM, compared with a *K*^i^ value of 50 nM for human LDH-M (LDH-A4) [18].

In previous work, three naphthalene compounds—NDCA （2,6-naphthalenedicarboxylic acid), DBHCA (1,6-dibromo-2-hydroxynapthalene 3-carboxylic acid), and DHNA (3,5-dihyddroxy 2-napthoic acid)—featuring a gossypol core structure—were screened for their inhibitory effects on LDH activity in *B. microti*. Among these, DBHCA and DHNA demonstrated IC_50_ values of 52.7 μM and 30 μM, respectively, identifying them as promising lead compounds [19]. With the advancement of computer technology, computer-aided drug design (CADD) has become increasingly prevalent in drug research and development [20, 21]. This approach not only reduces the cost of drug development significantly but also facilitates the synthesis of similar naphthalene carboxylic compounds through chemical methods [22].

In this study, computer-aided drug design (CADD) technology is employed to design and screen novel compounds targeting the catalytic activity of BmLDH, using the lead compound DHNA as a foundation. The identified compounds were subsequently evaluated through surface plasmon resonance (SPR) analysis, enzyme activity inhibition assays, in vitro growth inhibition tests, and cytotoxicity tests to determine their inhibitory efficacy. This research represents a significant advancement in the exploration and development of antiparasitic drugs.

## Methods

### Establishment of DHNA structural modification database

Based on the interactions between relevant amino acid residues in the active site of BmLDH and DHNA, modifications were made to DHNA by altering the positions of the carboxyl and hydroxyl groups and introducing various side chain groups. Discovery Studio software (v18.1.100.18065) was utilized to design small molecule structures and generate sets of these structures. The “Prepare Ligands” and “Minimize Ligands” modules were employed to refine and optimize the collections of structures.

### Molecular docking

The protein structure of BmLDH (PDB accession no. 6K13) was retrieved from the Protein Data Bank and preprocessed using Discovery Studio software. The resulting data were used as receptor data for subsequent analyses. Initially, LibDOCK was employed to dock the receptors and ligands, and the top 100 conformations were selected based on their scores. These small molecules, identified by their top LibDOCK scores, were then subjected to CDOCKER docking. In the flexible docking process, Arg99 was designated as a flexible residue, and the top 100 molecules were chosen based on their CDOCKER energy scores.

### Synthesis

Two compounds, TA and TB, were synthesized following the specified synthesis route (Additional file 1: Supplementary Fig. 1).

#### Synthesis of compound TA

Compound 2: to a cooled solution of compound 1 (19 g, 85.2 mmol, 1 eq) in *N*,*N*-dimethylformamide (DMF) (170 mL) at 0 ℃, triisopropylsilyl chloride (TIPSCl) (21.4 g, 110.7 mmol, 23.7 mL, 1.3 eq), imidazole (12.2 g, 178.9 mmol, 2.1 eq), and 4-dimethylaminopyridine (DMAP) (2.1 g, 17.0 mmol, 0.2 eq) were added. The reaction mixture was allowed to warm to 15–20 ℃ and stirred for 6 h.

Compound 3: flask 1: a portion (60 mL) of a solution of compound 2 (24 g, 63.23 mmol, 1 eq) in tetrahydrofuran (THF) (300 mL) was added to a flame-dried flask containing magnesium (1.5 g, 62.1 mmol, 0.98 eq) and iodine (160.6 mg, 632.6 μmol, 127.4 μL, 0.01 eq) under a nitrogen atmosphere. The mixture was heated to 65–70 ℃, and the remaining 240 mL of the bromo-compound was added dropwise at a rate to maintain reflux. After the addition was complete, the dark solution was stirred at 65–70 ℃ for 1 h, then cooled to 15–20 ℃. Flask 2: a solution of phthalic anhydride (9.4 g, 63.3 mmol, 1 eq) in THF (120 mL) was heated to 65–70 ℃. The solution from flask 1 was then added dropwise over 1 h and stirred at 65–70 ℃ for 4 h. The reaction mixture was cooled to 15–20 ℃ and quenched by the addition of 2 M HCl until the pH was adjusted to 2–3 at −10 to 0 ℃.

Compound 4: palladium on carbon (Pd/C, 1.1 g, 1.7 mmol, 10% purity) was added to a solution of compound 3 (11 g, 24.5 mmol, 1 eq) in acetic acid (AcOH, 110 mL) under a nitrogen atmosphere. The suspension was degassed under vacuum and purged with hydrogen several times. The mixture was stirred under hydrogen (40–50 psi) at 60–70 ℃ for 24 h. The mixture was stirred under hydrogen (40–50 psi) at 60–70 ℃ for an additional 6 h.

Compound target A (TA): TBAF (1 M, 20.9 mL, 1.3 eq) was added to a solution of compound 4 (7 g, 16.1 mmol, 1 eq) in THF (80 mL), and the mixture was stirred at 15–20 ℃ for 1 h.

#### Synthesis of compound TB

Compound 6: KOAc (8.6 g, 87.3 mmol, 2 eq), bis(pinacolato)diboron (11.1 g, 43.7 mmol, 1 eq), and Pd(dppf)Cl_2_ (1.3 g, 1.8 mmol, 0.04 eq) was added to a solution of compound 5 (10 g, 43.7 mmol, 1 eq) in 1,4-dioxane (100 mL) under a nitrogen atmosphere. The reaction mixture was stirred at 80–85 ℃ for 4 h.

Compound 7: compound 6 (8 g, 28.9 mmol, 1.4 eq), Pd(dppf)Cl_2_ (1.5 g, 2.1 mmol, 0.1 eq), and K_2_CO_3_ (14.6 g, 105.4 mmol, 5 eq) was added to a solution of compound 2 (8 g, 21.1 mmol, 1 eq) in 1,4-dioxane (80 mL) and H_2_O (19 mL) under a nitrogen atmosphere. The reaction mixture was stirred at 80–85 ℃ for 8 h.

Compound 8: TBAF (1 M, 27.4 mL, 1.5 eq) was added to a solution of compound 7 (8.2 g, 18.3 mmol, 1 eq) in THF (80 mL), and the mixture was stirred at 15–20 ℃ for 2 h.

Compound target B (TB): NaOH (2.7 g, 68.5 mmol, 2.5 eq) was added to a solution of compound 8 (8 g, 27.4 mmol, 1 eq) in MeOH (80 mL) and H_2_O (160 mL). The mixture was stirred at 15–20 ℃ for 3 h.

### Parasites

The *B. microti* ATCC PRA-99™ strain was supplied by the National Institute of Parasitic Diseases, Chinese Center for Disease Control and Prevention (Shanghai, China). This strain is preserved in liquid nitrogen with the addition of dimethyl sulfoxide (DMSO) at the National Key Laboratory of Agricultural Microbiology, Huazhong Agricultural University, China.

### Expression and purification of rBmLDH

The expression and purification of rBmLDH were conducted according to a previously established protocol [19]. Briefly, a bacterial culture of pET-28a-BmLDH/BL21 (DE3), stored at −80 ℃, was thawed and resuscitated. To induce rBmLDH expression, isopropylthio-β-d-galactoside (IPTG) was added, and the culture was incubated at 28 ℃. After sufficient induction, the cells were harvested. Recombinant BmLDH was then purified using a Ni affinity chromatography column (GE Healthcare, Uppsala, Sweden) following the manufacturer’s instructions.

### Surface plasmon resonance (SPR) assays

The binding properties of rBmLDH to small molecule compounds were assessed using a Biacore T200 system (GE Healthcare, Uppsala, Sweden) via surface plasmon resonance (SPR) assays. A CM5 sensor chip was inserted into the Biacore T200 system and flushed with PBST buffer (pH 7.5). To activate the sensor chip, a 1:1 (v/v) mixture of 0.1 M NHS and 0.4 M EDC was injected at a flow rate of 10 μL/min for 1200 s. Purified rBmLDH was diluted to a concentration of 25 μg/mL in 10 mM sodium acetate buffer (pH 4) and covalently immobilized onto the CM5 chip at a flow rate of 30 μL/min until the immobilization level reached 6100 RU. The reaction was halted by injecting ethanolamine. Novel small molecule compounds, initially prepared at a concentration of 100 mM, were further diluted in PBST to final concentrations of 20, 10, 5, 2.5, 1.25, and 0 μM. Flow cells 1 and 3 served as reference surfaces, while flow cells 2 and 4 were designated as experimental surfaces.

### Enzyme inhibition analysis

The TA and TB were dissolved in DMSO to create a 100 mM solution. The reaction system, conducted at 25 ℃, consisted of NaCO_3_ and NaHCO_3_ buffer (pH 9.5) with a total volume of 200 μL, containing 50 ng of rBmLDH, 1.5 mM NAD^+^, 10 mM L-lactic acid, and varying concentrations of the small molecule compounds. The increase in NADH production was monitored spectrophotometrically at OD_340_ (Optical density at 340 nm). The IC_50_ values for the new small molecule compounds were calculated on the basis of these results. The final concentration of DMSO did not affect rBmLDH activity, and all experiments were performed in triplicate.

### Inhibitory effect of TA and TB against the growth of *B. microti* in vitro

BALB/c mice were inoculated with 10^7^ *B. microti* (100 μL) via intraperitoneal injection. Once parasitemia reached 60%, blood was collected from the tails of the mice, and the initial percent parasitized erythrocytes (PPE) was diluted to approximately 0.8% using healthy mouse erythrocytes. TA and TB were prepared as 100 mM stock solutions and further diluted into concentration gradients of 500 μM, 250 μM, 125 μM, and 62.5 μM with growth medium (HL-1 supplemented with 10 μg/mg AlbuMax I, 1% HB101, 200 μM L-glutamine, 2% antibiotic/antimycotic 100× , and 20% fetal bovine serum). The growth medium without drug served as a negative control, while 10 μM diminazene aceturate (DA) was used as a positive control. Cultures were incubated for 72 h at 37 ℃ in a gas mixture of 2% O_2_, 5% CO_2_, and 93% N_2_ [23, 24]. After incubation, blood smears were prepared, stained with Giemsa, and the parasitemia rate was calculated. All experiments were performed in triplicate.

### Cytotoxicity test by CCK-8 method

The mycoplasma-free Vero cell line (ATCC-CCL-81, immortalized cells) was cryopreserved in liquid nitrogen with DMSO at the National Key Laboratory of Agricultural Microbiology, Huazhong Agricultural University, China. The cytotoxicity of compounds TA and TB was assessed in Vero cells using the CCK-8 assay (Vazyme). Briefly, Vero cells (100 µL, 10,000 cells/well) were cultured in Dulbecco’s modified Eagle medium (DMEM) supplemented with 10% fetal bovine serum (FBS) and 100 U/mL penicillin–streptomycin at 37 ℃ with 5% CO_2_ for 24 h. The cells were then treated with varying concentrations of TA (2000, 1000, 500, 250, and 125 μM) or TB (800, 400, 200, 100, and 50 μM) for 72 h. After the treatment period, the medium containing TA and TB was replaced with fresh medium. Subsequently, 10 µL of CCK-8 solution was added to each well, and the plate was incubated for an additional 3 h. Absorbance was measured at 450 nm using a multifunctional enzyme marker (Envision, PE, USA). Stock solutions of TA and TB were prepared at a concentration of 100 mM in absolute ethanol. Doxorubicin hydrochloride at 40 µM served as the positive control, while 2% absolute ethanol served as the negative control. The concentration required to achieve 50% cytotoxicity (CC_50_) values were calculated using GraphPad Prism 8, and all cytotoxicity assays were performed in triplicate.

## Results

### Two small molecule compounds, TA and TB, were obtained through molecular docking screening and chemical synthesis

**Fig. 1:**
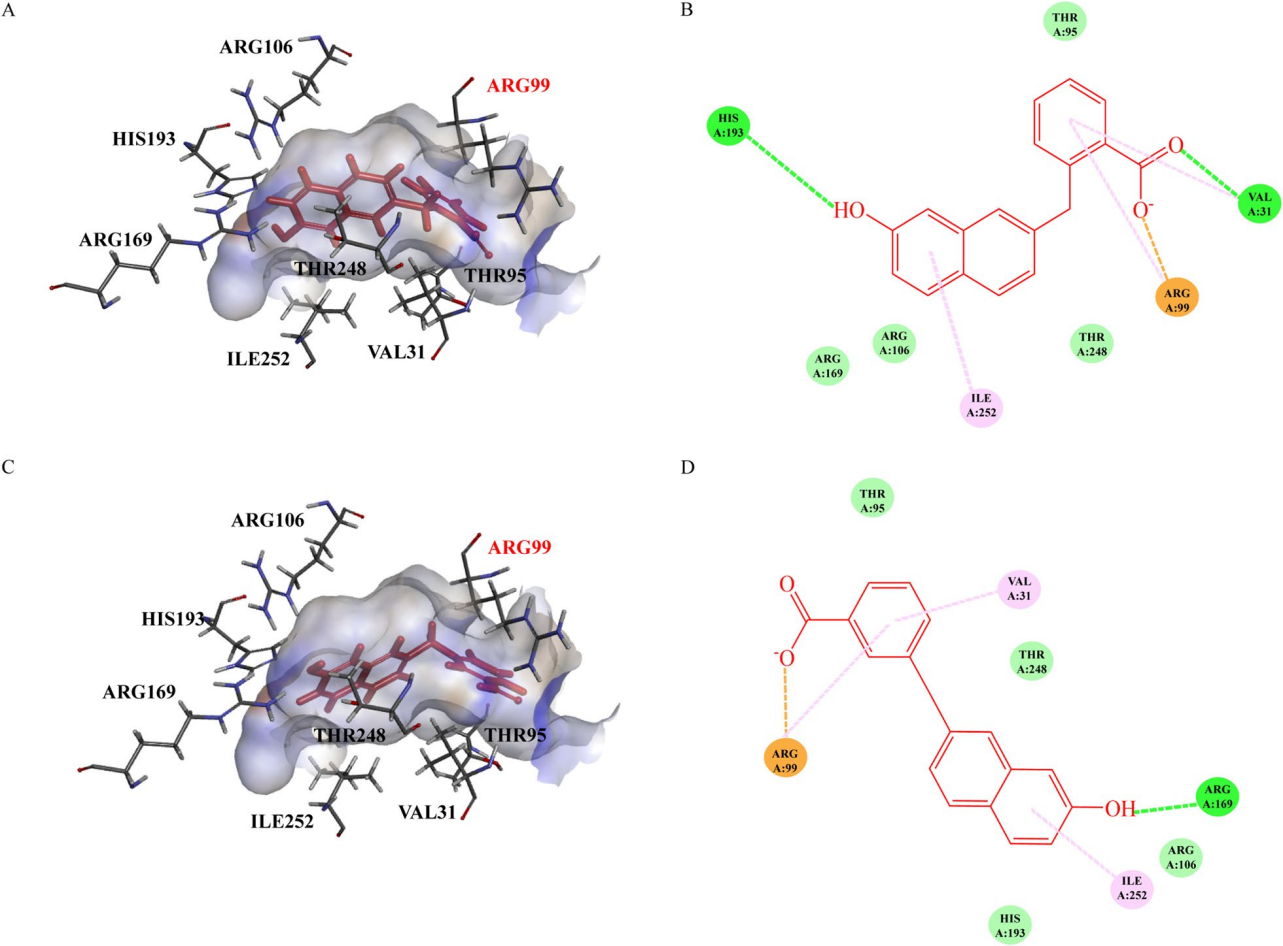
Results of molecular docking. **A** Molecular model of compound TA in complex with *Babesia microti* lactate dehydrogenase (BmLDH). **B** Interactions of TA with amino acid residues. **C** Molecular model of compound TB in complex with *Babesia microti* lactate dehydrogenase (BmLDH). **D** Interactions of TB with amino acid residues. Conventional hydrogen bonds are indicated by green dashed lines, Pi-alkyl bonds by pinkish-purple dashed lines, attractive charge interactions by orange dashed lines, and van der Waals interactions by light green dashed lines. The structure of the compounds is highlighted in red

After three rounds of molecular docking, five inhibitors that interacted with the Arg99 residue were selected from a modified DHNA compound database based on their docking scores (Additional file 1: Supplementary Table 1). The binding affinities of these screened small molecule compounds to the receptor were simulated, and the interactions between the ligands and the receptor were thoroughly analyzed. Among the five compounds, the structures of TA and TB demonstrated strong interactions with Arg99 and exhibited a greater degree of spatial complementarity with the receptor’s active sites (Fig. 1), the docking score of TA, TB and DHNA are presented in Table 1. The chemical synthesis of TA and TB was also relatively straightforward. Owing to their favorable properties, TA and TB were selected for further chemical synthesis.

**Table 1:** The docking score of TA, TB, and DHNA

Compound ID	Structure	CDOCKER energy
TA		−36
TB		−43.8
DHNA		−28.33

### Expression of the recombinant BmLDH

The recombinant protein was successfully expressed in *Escherichia coli* and purified using a Ni affinity column. The molecular weight of the purified recombinant protein was estimated to be approximately 37 kDa (Additional file 1: Supplementary Fig. 2). The purified protein was then employed in subsequent assays, including SPR and enzyme activity analysis.

### Both TA and TB can bind to rBmLDH

**Fig. 2:**
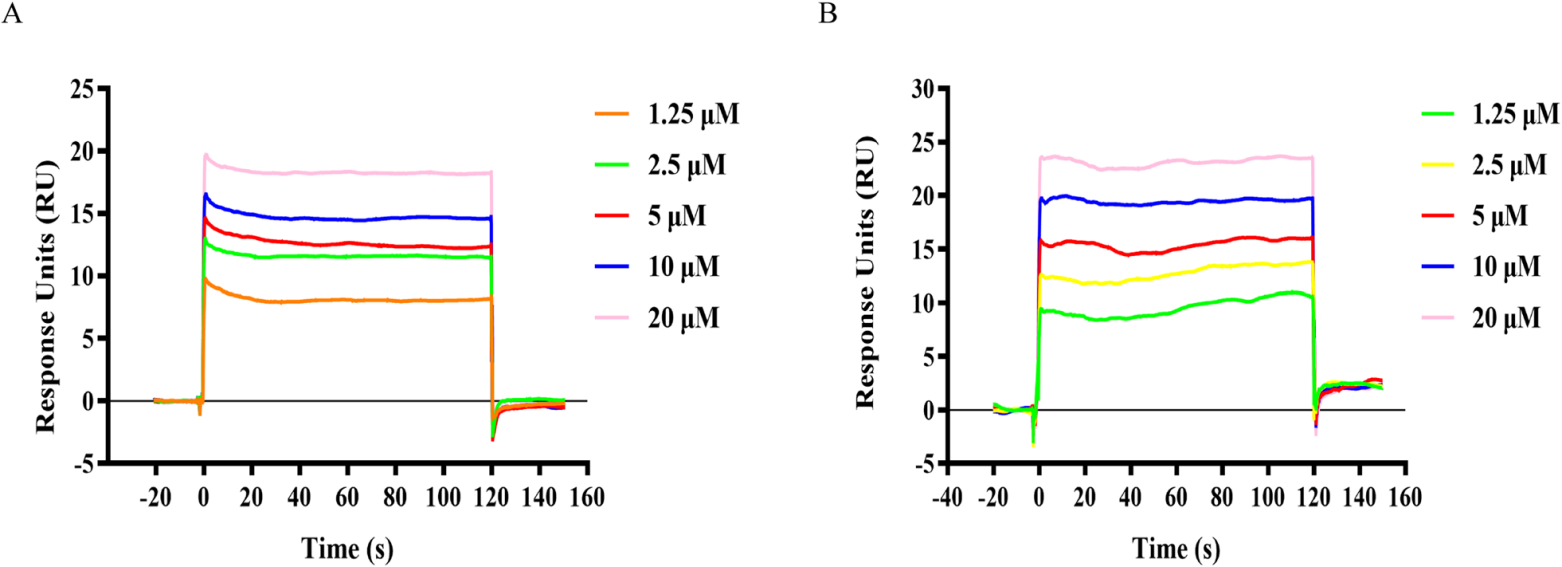
Surface plasmon resonance (SPR) kinetic sensorgrams. Binding of TA (**A**) and TB (**B**) to *B. microti* lactate dehydrogenase (BmLDH) at varying concentrations (20, 10, 5, 2.5, and 1.25 μM). Responses were reference subtracted and blank corrected

To evaluate the binding affinity of compounds TA and TB to recombinant *B. microti* lactate dehydrogenase (rBmLDH), we employed surface plasmon resonance (SPR) analysis with varying concentrations of each compound. The binding data was analyzed using a 1:1 binding kinetics model, yielding valuable insights into their binding dynamics (Fig. 2). The equilibrium dissociation constant (*K*d) was calculated as 11.3 × 10^–6^ M for compound TA (Fig. 2A) and 13.2 × 10^–6^ M for compound TB (Fig. 2B). Notably, TB exhibited a marginally higher binding affinity for rBmLDH relative to TA. Furthermore, when compared with the lead compound (DHNA), both TA and TB demonstrated over a threefold increase in binding capacity to rBmLDH. These findings suggest that the structural modifications made in compounds TA and TB have significantly amplified their binding capabilities to rBmLDH.

### Both TA and TB can inhibit the catalytic activity of the rBmLDH

**Fig. 3:**
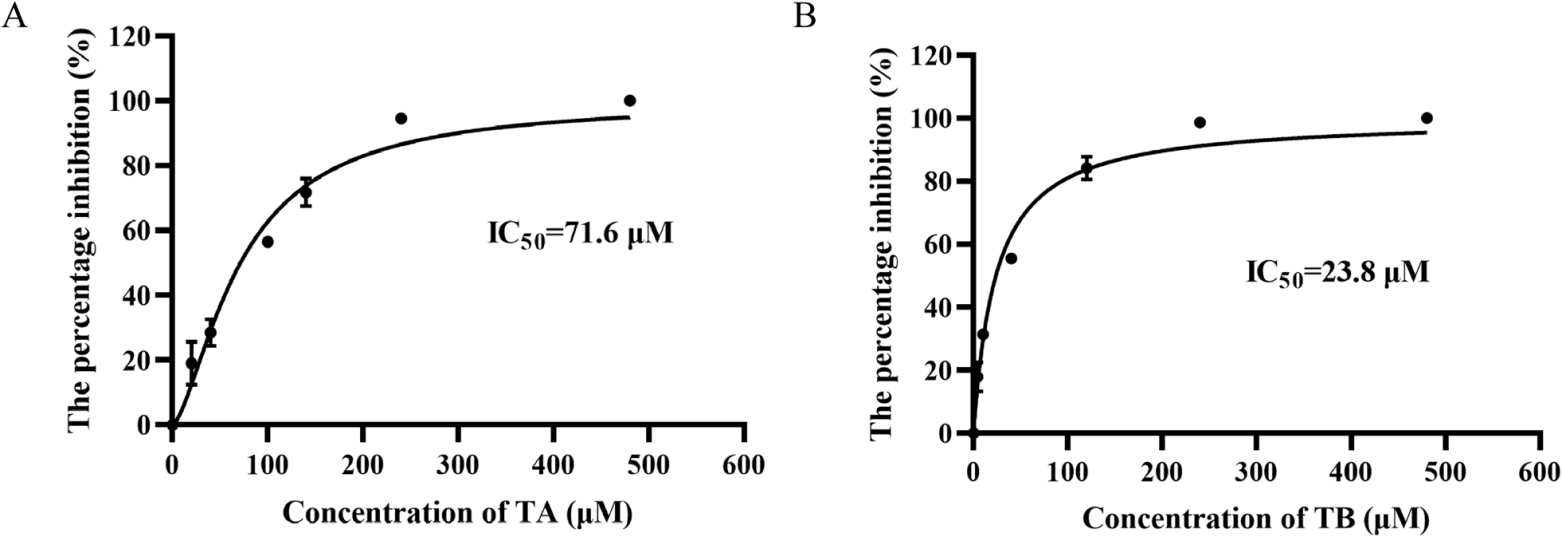
Inhibitory effects of TA and TB on rBmLDH. **A** The impact of various concentrations of compound TA on recombinant proteins. **B** The effect of varying concentrations of compound TB on recombinant proteins

To further investigate the functional implications of the binding affinity, we evaluated the inhibitory effects of TA and TB on rBmLDH activity (Fig. 3). The half-maximal inhibitory concentration (IC_50_) values were established, revealing that compound TB inhibited rBmLDH activity with an IC_50_ of 23.8 μM (Fig. 3B), while compound TA exhibited an IC_50_ of 71.6 μM (Fig. 3A). These results indicate that TB possesses a more substantial inhibitory effect on rBmLDH compared with TA. However, it is essential to note that TB’s inhibitory efficacy was not as pronounced as that of the lead compound, DHNA.

### Lactate dehydrogenase inhibitors interfere with the growth of *B. microti* in vitro

**Fig. 4:**
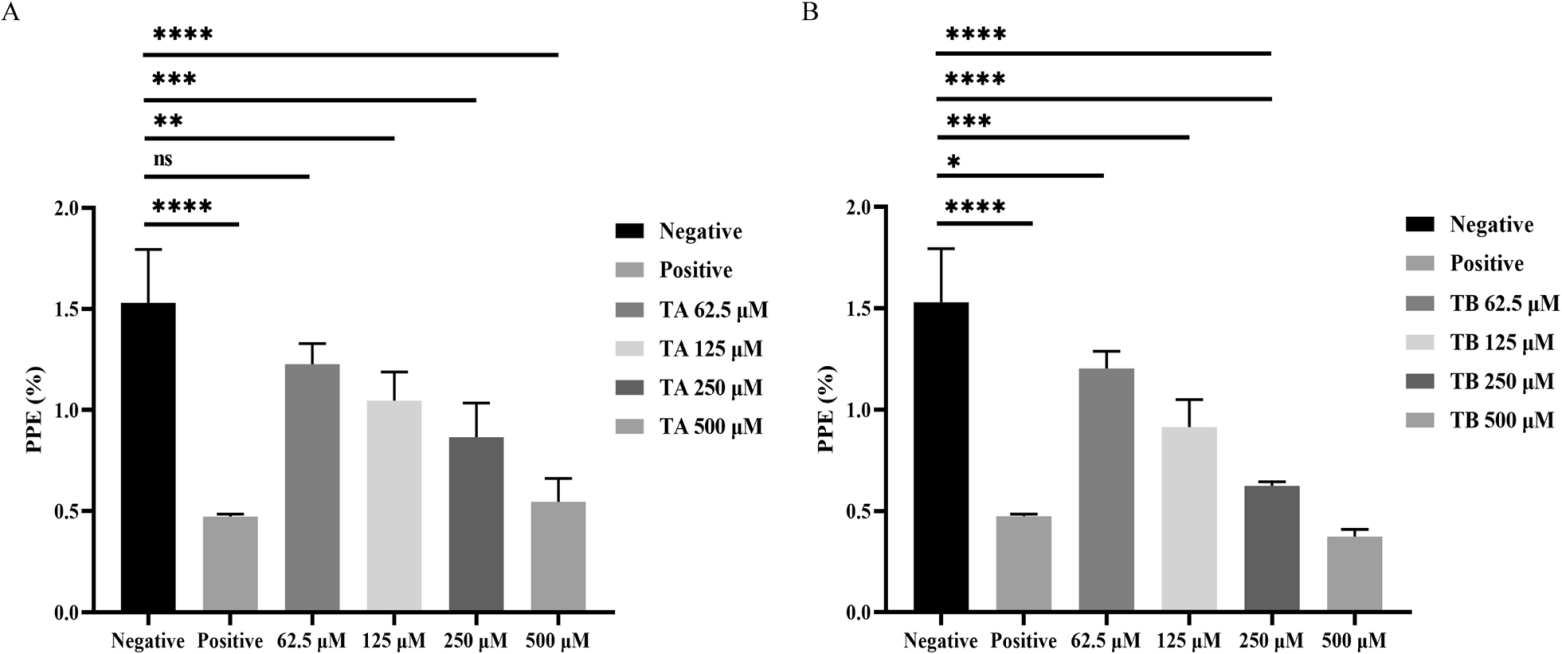
Growth inhibition analysis of *B. microti* by compounds TA and TB. **A** The inhibitory effect of different concentrations of compound TA. **B** The inhibitory effect of varying concentrations of compound TB. In vitro growth rates were assessed in the presence of different concentrations of TA and TB. Diminazene aceturate (DA) at 10 μM was used as a positive control. Asterisks denote a significant difference between the treatment and control groups (four asterisks indicate *P* < 0.0001). Error bars represent the mean ± SD (*n* = 3 independent experiments), and the chart was generated using GraphPad Prism 8. “Ns” indicates no significant difference

Both compounds were evaluated for their antiparasitic effects on *B. microti*in vitro. The IC_50_ values for growth inhibition were determined to be 121.7 μM for TA and 111.7 μM for TB (Fig. 4). Again, TB outperformed TA in terms of inhibitory efficacy. Nonetheless, the inhibitory action of both compounds was less potent than that of DHNA in the in vitro parasite inhibition assessment.

### Both TA and TB inhibit the growth of Vero cells

**Fig. 5:**
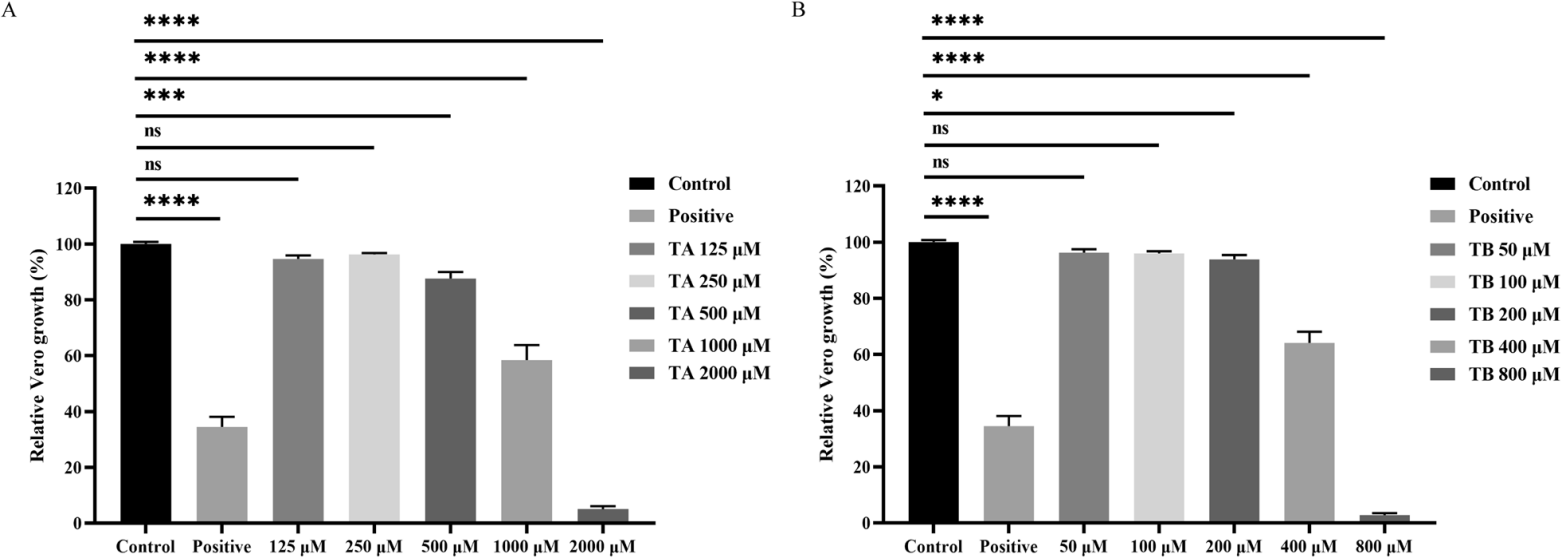
Cytotoxicity of compounds TA and TB against the Vero cell line. **A** Assessment of various concentrations (2000, 1000, 500, 250, and 125 μM) of TA on Vero cells *in vitro*. Cell viability was measured using the CCK-8 method at 72 h post treatment. **B** Evaluation of different concentrations (800, 400, 200, 100, and 50 μM) of TB against Vero cells *in vitro*. Doxorubicin hydrochloride at 40 μM served as the positive control. Cytotoxic effects on Vero cells were evaluated using the CCK-8 assay at 72 h post treatment. Relative growth rates of cells were statistically analyzed by one-way analysis of variance (ANOVA), with asterisks indicating significant differences between the drug-treated groups and the negative control (four asterisks represent *P* < 0.0001). Error bars represent the mean ± SD (*n* = 3 independent experiments), and all charts were created using GraphPad Prism 8. “Ns” indicates no significant difference

To assess the potential for therapeutic use, the cytotoxicity of the compounds was evaluated on Vero cells, and the selection index (SI) was computed. The CC_50_ values were found to be 1030 μM for TA and 434.4 μM for TB (Fig. 5). Consequently, the selection indices were calculated to be 8.5 for TA and 3.9 for TB (Table 2). Although both TA and TB displayed significant inhibition of rBmLDH catalytic activity and affected *B. microti* growth in vitro, their practical application is constrained by their relatively low selection indices, highlighting a need for further optimization to enhance their therapeutic potential.

**Table 2:** In vitro evaluation of compounds TA and TB against the asexual blood stage of *B. microti*, cytotoxicity in Vero cells, and selection index (SI)

Structure	Compound ID	BmLDH IC_50_ (μM)	*B. microti* IC_50_ (μM)	Vero CC_50_ (μM)	SI
	TA	71.6	121.7	1030	8.5
	TB	23.8	111.7	434.4	3.9

## Discussion

The continuous use of pharmaceutical agents often leads to the development of drug resistance—a phenomenon observed with commonly used clinical drugs for the treatment of babesiosis, such as atovaquone, quinine, azithromycin, and clindamycin, all of which exhibit varying degrees of resistance [11, 25]. Consequently, there is an urgent need for the development of novel therapeutic interventions [26].

However, drug development poses significant challenges, with the identification of potential drug targets being crucial for structure-based drug design. Given the unique parasitic environment of *Babesia*, the lactate dehydrogenase (LDH)-mediated glycolytic pathway plays a vital role in providing ATP for the parasite’s growth. The importance of LDH has been established in various apicomplexan protozoa [27, 28]. Over 50 compounds structurally similar to NADH have been screened through molecular docking, exploring their potential as antimalarial agents [29]. Similarly, a series of 1,2,3-triazole derivatives have been identified via molecular docking that share structural similarity with NADH and can bind to the active site of LDH, thereby exhibiting antimalarial effects [30]. These findings suggest that LDH is a promising target for drug development, and designing small-molecule drugs based on the structure of LDH’s natural substrates represents an effective strategy. Therefore, molecular docking studies can be a useful tool in the process of discovering and designing new drugs.

Gossypol has shown inhibitory effects on recombinant LDH proteins and has demonstrated the ability to suppress the growth of *Babesia* when added as a supplement during in vitro cultivation [14, 19, 31, 32]. Currently, several gossypol derivatives, characterized by substituted naphthalene cores containing hydroxyl, aldehyde, or carboxylic acid groups at different positions, have shown promising biological activities [33]. Therefore, modifying the gossypol structure may serve as a viable strategy for drug design [34, 35]. In our previous study, the complex structure of LDH with oxalate was determined [19]. However, its toxicity has limited its clinical applications. This study focuses on gossypol derivatives, revealing that oxalate binds at the same site as pyruvate within the protein’s binding site, suggesting that oxalate may also inhibit the protein’s activity, albeit weakly [13, 32]. On the basis of the crystal structure of LDH, three gossypol derivatives (DHNA, NDCA, and DBHCA) were screened through molecular docking and further modified on the basis of DHNA [19].

Research into the interactions between gossypol derivatives and the active site of LDH has revealed that hydroxyl and carboxylic acid substituents are key determinants for interaction with residues at this active site [33]. Consequently, two compounds with differences in the position of the carboxyl group were identified. Both compounds demonstrated rapid binding and dissociation kinetics in surface plasmon resonance (SPR) experiments. Rapid binding allows for quick drug action, while rapid dissociation ensures drug safety, although it may also impact drug efficacy.

Although the *K*_D_ values of the two compounds were similar according to the SPR results, the IC_50_ values of compounds TA and TB in the enzyme activity inhibition test showed significant differences. This discrepancy may be attributed to variations in the compounds’ ability to form hydrogen bonds with specific amino acid residues. Attempts were made to crystallize the compounds with the recombinant protein; unfortunately, we were unable to obtain the complex structure.

Furthermore, the SPR results indicated that compounds TA and TB exhibited stronger binding affinities to rBmLDH than DHNA [19]. However, this does not necessarily imply that TA and TB are superior to DHNA in inhibiting rBmLDH. The enzyme activity inhibition test and the in vitro growth inhibition assay of *B. microti* confirmed this. Although TA and TB exhibited more than three times higher affinity for rBmLDH than DHNA, their inhibitory effects on rBmLDH activity and the growth of *B. microti* in vitro did not correlate with the SPR results.

Indeed, an ideal drug should possess both high affinity (fast binding) and a favorable duration of binding (slow dissociation). In this case, the observed phenomenon could be attributed to the rapid binding and rapid dissociation process between the two compounds and rBmLDH. Rapid binding contributes to high affinity, but rapid dissociation may lead to weaker inhibitory effects [36].

Currently, we have successfully modified and obtained two small-molecule compounds based on DHNA using computer-aided drug design techniques. In light of the SPR results, our goal is to further refine the structures of compounds TA and TB to enhance their affinity for the recombinant protein and reduce the dissociation rate. This approach aims to prolong the drug’s efficacy by achieving a more favorable binding profile.

## Conclusions

In summary, both compounds exhibit binding affinity to recombinant *Babesia microti* lactate dehydrogenase (rBmLDH) and demonstrate inhibitory effects on the growth of *B. microti* in vitro. However, there remains significant potential for improving their efficacy against *B. microti*. Cytotoxicity tests conducted in Vero cells indicate that compound TA has a higher selectivity index compared with compound TB. The surface plasmon resonance (SPR) results will guide future modifications aimed at enhancing the binding affinity of these compounds. Additionally, the selectivity of these compounds for rBmLDH over human proteins is lower than that of the lead compound DHNA, underscoring the need for further optimization. Overall, these findings provide valuable insights for the design of new drugs with similar structural features.

## Supplementary Information

Additional file 1: Supplementary Fig. 1. Synthesis of TA and TBa. Supplementary Fig. 2. SDS–PAGE of 486 prokaryotic expression stained by Coomassie blue.

## Data Availability

No datasets were generated or analysed during the current study.

## Abbreviations

RBC: Red blood cell
TCA cycle: Tricarboxylic acid cycle
LDH: Lactate dehydrogenase
DHNA: 3,5-Dihydroxy-2-naphthoxylic acid
TA: Target A
TB: Target B
SPR: Surface plasmon resonance
*B. microti*: *Babesia microti*
*K*_D_: Dissociation constant
MDH: Malate dehydrogenase
DMF: *N*,*N*-dimethylformamide
TIPSCl: Triisopropylsilyl chloride
DMAP: 4-Dimethylaminopyridine
TLC: Thin-layer chromatography
MTBE: Methyl *tert*-butyl ether
THF: Tetrahydrofuran
TBAF: Tetrabutylammonium fluoride
nm: Nanometer
DA: Diminazene aceturate
IPTG: Isopropylthio-β-d-galactoside
mL: Milliliter
μM: µMol/L, micromole per liter
CADD: Computer aided drug design
PPE: Percent of parasitized erythrocytes

## References

[1] Herwaldt BL, Linden JV, Bosserman E, Young C, Olkowska D, Wilson M. Transfusion-associated Babesiosis in the united states: a description of cases. Ann Intern Med. 2011;155:509–19. 10.7326/0003-4819-155-8-201110180-00362. 21893613 10.7326/0003-4819-155-8-201110180-00362

[2] de Ramón C, Cid J, Rodríguez-Tajes S, Álvarez-Martínez MJ, Valls ME, Fernández J, et al. Severe *Babesia microti* infection in an American immunocompetent patient diagnosed in Spain. Transfus Apheres Sci. 2016;55:243–4. 10.1016/j.transci.2016.07.021. 10.1016/j.transci.2016.07.021 27499182

[3] Bakkour S, Chafets DM, Wen L, Muench MO, Telford SR 3rd, Erwin JL, et al. Minimal infectious dose and dynamics of *Babesia microti* parasitemia in a murine model. Transfusion. 2018;58:2903–10. 10.1111/trf.14889. 30264498 10.1111/trf.14889

[4] Goethert HK, Molloy P, Berardi V, Weeks K, Telford SR. Zoonotic Babesia microti in the northeastern U.S.: evidence for the expansion of a specific parasite lineage. PLoS ONE. 2018;13:0193837. 10.1371/journal.pone.0193837. 10.1371/journal.pone.0193837 PMC5864094 29565993

[5] Kumar A, Oryan J, Krause PJ, et al. The global emergence of human Babesiosis. Pathogens. 2021;10:1447. 10.3390/pathogens10111447. 34832603 10.3390/pathogens10111447 PMC8623124

[6] Kletsova EA, Spitzer ED, Fries BC, Marcos LA. Babesiosis in Long Island: review of 62 cases focusing on treatment with azithromycin and atovaquone. Ann Clin Microbiol Antimicrob. 2017;16:26. 10.1186/s12941-017-0198-9. 28399851 10.1186/s12941-017-0198-9 PMC5387270

[7] Krause PJ, Lepore T, Sikand VK, Gadbaw J, Burke G, Telford SR, et al. Atovaquone and azithromycin for the treatment of Babesiosis. N Engl J Med. 2000;343:1454–8. 10.1056/nejm200011163432004. 11078770 10.1056/NEJM200011163432004

[8] Lemieux JE, Tran AD, Freimark L, Schaffner SF, Goethert H, Andersen KG, et al. A global map of genetic diversity in *Babesia microti* reveals strong population structure and identifies variants associated with clinical relapse. Nat Microbiol. 2016;1:16079. 10.1038/nmicrobiol.2016.79. 27572973 10.1038/nmicrobiol.2016.79 PMC5563076

[9] Simon MS, Westblade LF, Dziedziech A, Visone JE, Furman RR, Jenkins SG, et al. Clinical and molecular evidence of atovaquone and azithromycin resistance in relapsed *Babesia microti* infection associated with rituximab and chronic lymphocytic Leukemia. Clin Infect Dis. 2017;65:1222–5. 10.1093/cid/cix477. 28541469 10.1093/cid/cix477 PMC6248624

[10] Lawres LA, Garg A, Kumar V, Bruzual I, Forquer IP, Renard I, et al. Radical cure of experimental babesiosis in immunodeficient mice using a combination of an endochin-like quinolone and atovaquone. J Exp Med. 2016;213:1307–18. 10.1084/jem.20151519. 27270894 10.1084/jem.20151519 PMC4925016

[11] Renard I, Ben MC. Treatment of human Babesiosis: then and now. Pathogens. 2021;10:1120. 10.3390/pathogens10091120. 34578153 10.3390/pathogens10091120 PMC8469882

[12] Cornillot E, Hadj-Kaddour K, Dassouli A, Noel B, Ranwez V, Vacherie B, et al. Sequencing of the smallest Apicomplexan genome from the human pathogen *Babesia vitro*. Nucleic Acids Res. 2012;40:9102–14. 10.1093/nar/gks700. 22833609 10.1093/nar/gks700 PMC3467087

[13] Yu L, Shen Z, Liu Q, Zhan X, Luo X, An X, et al. Crystal structures of *Babesia microti* lactate dehydrogenase BmLDH reveal a critical role for Arg99 in catalysis. FASEB J. 2019;33:13669–82. 10.1096/fj.201901259R. 31585506 10.1096/fj.201901259R

[14] He L, Bastos RG, Yu L, Laughery JM, Suarez CE. Lactate dehydrogenase as a potential therapeutic drug target to control *Babesia bigemina*. Front Cell Infect Microbiol. 2022. 10.3389/fcimb.2022.870852. 35521220 10.3389/fcimb.2022.870852 PMC9062099

[15] Doherty JR, Cleveland JL. Targeting lactate metabolism for cancer therapeutics. J Clin Investig. 2013;123:3685–92. 10.1172/jci69741. 23999443 10.1172/JCI69741 PMC3754272

[16] Lu Y, Li J, Dong C-E, Huang J, Zhou H-B, Wang W. Recent advances in gossypol derivatives and analogs: a chemistry and biology view. Future Med Chem. 2017;9:1243–75. 10.4155/fmc-2017-0046. 28722469 10.4155/fmc-2017-0046

[17] Saifee NH, Krause PJ, Wu Y. Apheresis for babesiosis: therapeutic parasite reduction or removal of harmful toxins or both? J Clin Apheresis. 2015;31:454–8. 10.1002/jca.21429. 26481763 10.1002/jca.21429

[18] Deck LM, Royer RE, Chamblee BB, Hernandez VM, Malone RR, Torres JE, et al. Selective inhibitors of human lactate dehydrogenases and lactate dehydrogenase from the malarial parasite *Plasmodium falciparum*. J Med Chem. 1998;41:3879–87. 10.1021/jm980334n. 9748363 10.1021/jm980334n

[19] Yu L, Zhan X, Liu Q, Sun Y, Li M, Zhao Y, et al. Identifying the naphthalene-based compound 3,5-dihydroxy 2-napthoic acid as a novel lead compound for designing lactate dehydrogenase-specific antibabesial drug. Front Pharmacol. 2020. 10.3389/fphar.2019.01663. 32116673 10.3389/fphar.2019.01663 PMC7025647

[20] Liu S, Alnammi M, Ericksen SS, Voter AF, Ananiev GE, Keck JL, et al. Practical model selection for prospective virtual screening. J Chem Inf Model. 2019;59:282–93. 10.1021/acs.jcim.8b00363. 30500183 10.1021/acs.jcim.8b00363 PMC6351977

[21] Maia EHB, Assis LC, de Oliveira TA, da Silva AM, Taranto AG. Structure-based virtual screening: from classical to artificial intelligence. Front Chem. 2020. 10.3389/fchem.2020.00343. 32411671 10.3389/fchem.2020.00343 PMC7200080

[22] Macalino SJY, Gosu V, Hong S, Choi S. Role of computer-aided drug design in modern drug discovery. Arch Pharmacal Res. 2015;38:1686–701. 10.1007/s12272-015-0640-5. 10.1007/s12272-015-0640-5 26208641

[23] Wang S, Li M, Luo X, et al. Inhibitory effects of fosmidomycin against Babesia microti in vitro. Front Cell Dev Biol. 2020;8:247. 10.3389/fcell.2020.00247. 32411701 10.3389/fcell.2020.00247 PMC7198706

[24] Guo J, Luo X, Wang S, He L, Zhao J. Xanthohumol and gossypol are promising inhibitors against Babesia microti by In Vitro culture via high-throughput screening of 133 natural products. Vaccines (Basel). 2020;8:613. 10.3390/vaccines8040613. 33081295 10.3390/vaccines8040613 PMC7711813

[25] Ji S, Rizk MA, Galon EM, El-Alfy E-S, Mizukawa Y, Kojima M, et al. Anti-babesial activity of a series of 6,7-dimethoxyquinazoline-2,4-diamines (DMQDAs). Acta Trop. 2024;249:107069. 10.1016/j.actatropica.2023.107069. 37952866 10.1016/j.actatropica.2023.107069

[26] Wormser GP, Prasad A, Neuhaus E, Joshi S, Nowakowski J, Nelson J, et al. Emergence of resistance to Azithromycin-atovaquone in immunocompromised patients with *Babesia microti* infection. Clin Infect Dis. 2010;50:381–6. 10.1086/649859. 20047477 10.1086/649859

[27] Choi S-R, Pradhan A, Hammond NL, Chittiboyina AG, Tekwani BL, Avery MA. Design, synthesis, and biological evaluation of *Plasmodium falciparum* lactate dehydrogenase inhibitors. J Med Chem. 2007;50:3841–50. 10.1021/jm070336k. 17636950 10.1021/jm070336k

[28] Abdelbaset AE, Fox BA, Karram MH, Ellah MRA, Bzik DJ, Igarashi M. Lactate dehydrogenase in *Toxoplasma gondii* controls virulence, bradyzoite differentiation, and chronic infection. PLoS ONE. 2017;12:0173745. 10.1371/journal.pone.0173745. 10.1371/journal.pone.0173745 PMC5360243 28323833

[29] Rodrigues MM, Penna-Coutinho J, Cortopassi WA, Oliveira AA, França TCC, Krettli AU. Antimalarial activity of potential inhibitors of *Plasmodium falciparum* lactate dehydrogenase enzyme selected by docking studies. PLoS ONE. 2011. 10.1371/journal.pone.0021237. 21779323 10.1371/journal.pone.0021237 PMC3136448

[30] Valério Lopes F, Fazza Stroppa PH, Marinho JA, Reis Soares R, de Azevedo AL, Capriles Goliatt PVZ, et al. 1,2,3-Triazole derivatives: synthesis, docking, cytotoxicity analysis and in vivo antimalarial activity. Chemico-Biol Interact. 2021. 10.1016/j.cbi.2021.109688. 10.1016/j.cbi.2021.109688 34627786

[31] Bork S, Okamura M, Boonchit S, Hirata H, Yokoyama N, Igarashi I. Identification of *Babesia bovis* l-lactate dehydrogenase as a potential chemotherapeutical target against bovine babesiosis. Mol Biochem Parasitol. 2004;136:165–72. 10.1016/j.molbiopara.2004.03.009. 15478796 10.1016/j.molbiopara.2004.03.009

[32] Yu L, Liu Q, Luo W, Zhao J, Alzan HF, He L. The structural basis of *Babesia orientalis* lactate dehydrogenase. Front Cell Infect Microbiol. 2022. 10.3389/fcimb.2021.790101. 35071043 10.3389/fcimb.2021.790101 PMC8766848

[33] Conners R, Schambach F, Read J, Cameron A, Sessions RB, Vivas L, et al. Mapping the binding site for gossypol-like inhibitors of *Plasmodium falciparum* lactate dehydrogenase. Mol Biochem Parasitol. 2005;142:137–48. 10.1016/j.molbiopara.2005.03.015. 15978953 10.1016/j.molbiopara.2005.03.015

[34] Razakantoanina V, Phung NKP, Jaureguiberry G. Antimalarial activity of new gossypol derivatives. Parasitol Res. 2000;86:665–8. 10.1007/PL00008549. 10952267 10.1007/pl00008549

[35] Royer RE, Deck LM, Campos NM, Hunsaker LA, Vander Jagt DL. Biologically active derivatives of gossypol: synthesis and antimalarial activities of peri-acylated gossylic nitriles. J Med Chem. 1986;29:1799–801. 10.1021/jm00159a043. 3528492 10.1021/jm00159a043

[36] Rai G, Brimacombe KR, Mott BT, Urban DJ, Hu X, Yang S-M, et al. Discovery and optimization of potent, cell-active Pyrazole-based inhibitors of lactate dehydrogenase (LDH). J Med Chem. 2017;60:9184–204. 10.1021/acs.jmedchem.7b00941. 29120638 10.1021/acs.jmedchem.7b00941 PMC5894102

