# Supplementary material for: Design and screening of novel molecular compounds targeting lactate dehydrogenase of Babesia microti
Source: Parasit Vectors. 2025 Feb 23;18:69. doi: 10.1186/s13071-024-06623-9 (PMC11847361; doi:10.1186/s13071-024-06623-9)
Supplement: Supplementary file 1 — Additional file 1: Supplementary Fig. 1. Synthesis of TA and TBa. Supplementary Fig. 2. SDS–PAGE of 486 prokaryotic expression stained by Coomassie blue. [file 13071_2024_6623_MOESM1_ESM.docx]

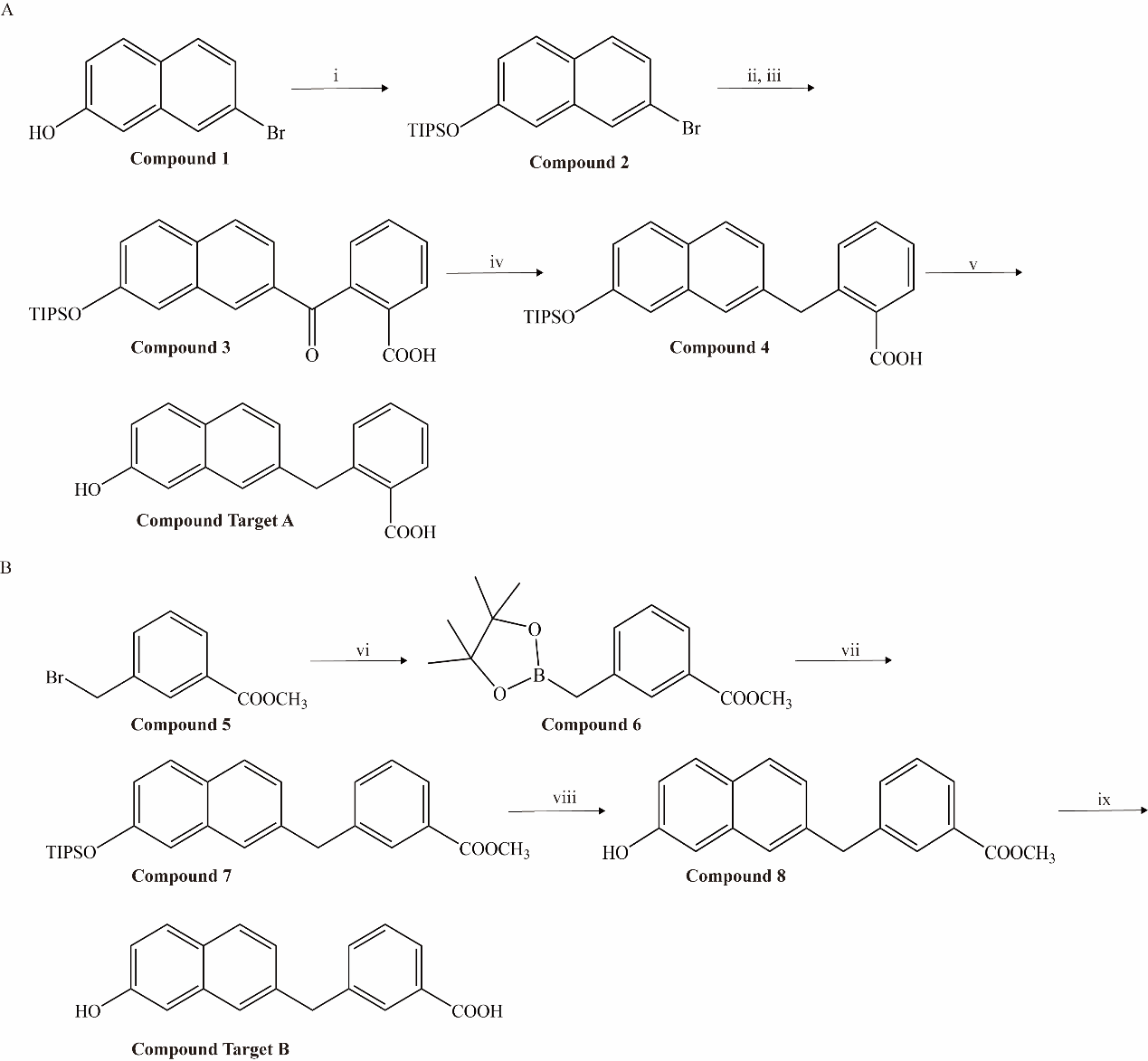


Fig. S1 Synthesis of TA (**A**) and TB^a^(**B**).

^a^Reagents and conditions: (i) TIPSCL, imidazole, DMF, 15-20 ℃; (ii) Mg power, I_2_, THF, 65-70 ℃; (iii) phthalic anhydride, THF, 65-70 ℃; (iv) Pd/C, H_2_ (psi), AcOH, 60-70 ℃; (v) TABF, THF, rt; (vi) methyl 3-(bromomethyl)benzoate, Bis(pinacolato)diboron, Pd(dppf)Cl_2_, KOAc, 1, 4-dioxane, 80 ℃; (vii) compound 2, 1, 4-dioxane, H_2_O, Pd(dppf)Cl_2_, K_2_CO_3_, 80 ℃; (viii) TABF, THF, rt; (ix) NaOH (4M), HCl (2M), EtOH, H_2_O, rt.

***Synthesis of compound TA***

Compound 2: To a cooled solution of compound 1 (19 g, 85.2 mmol, 1 eq) in DMF (170 mL) at 0 ℃, TIPSCl (21.4 g, 110.7 mmol, 23.7 mL, 1.3 eq), imidazole (12.2 g, 178.9 mmol, 2.1 eq), and DMAP (2.1 g, 17.0 mmol, 0.2 eq) were added. The reaction mixture was allowed to warm to 15-20 ℃ and stirred for 6 hours. Then, the mixture was diluted with H_2_O (500 mL) and extracted with MTBE (300 mL, 100 mL × 3). The combined organic layers were washed with H_2_O (400 mL), dried over Na_2_SO_4_, filtered, and concentrated under reduced pressure to yield a residue. The residue was purified by column chromatography (SiO_2_, Petroleum ether/Ethyl acetate = 1:0). Compound 2 (29.0 g, 76.4 mmol, 89.7% yield) was obtained as a colorless oil and confirmed by ^1^HNMR. Compound 2: ^1^HNMR (400 MHz, CDCl_3_) δ ppm 1.16 (d, J=7.6 Hz, 18 H) 1.3 - 1.4 (m, 3 H) 7.1 - 7.2 (m, 2 H) 7.4 (dd, J=8.4, 1.6 Hz, 1 H) 7.6 (d, J=8.4 Hz, 1 H) 7.7 (d, J=9.6 Hz, 1 H) 7.9 (d, J=1.6 Hz, 1 H).

Compound 3: Flask 1: A portion (60 mL) of a solution of compound 2 (24 g, 63.23 mmol, 1 eq) in THF (300 mL) was added to a flame-dried flask containing magnesium (1.5 g, 62.1 mmol, 0.98 eq) and iodine (160.6 mg, 632.6 μmol, 127.4 μL, 0.01 eq) under a nitrogen atmosphere. The mixture was heated to 65-70 ℃, and the remaining 240 mL of the bromo compound was added dropwise at a rate to maintain reflux. After the addition was complete, the dark solution was stirred at 65-70 ℃ for 1 hour, then cooled to 15-20 ℃. Flask 2: A solution of phthalic anhydride (9.4 g, 63.3 mmol, 1 eq) in THF (120 mL) was heated to 65-70 ℃. The solution from Flask 1 was then added dropwise over 1 hour and stirred at 65-70 ℃ for 4 hours. The reaction mixture was cooled to 15-20 ℃ and quenched by the addition of 2 M HCl until the pH was adjusted to 2-3 at -10 to 0 ℃. The mixture was then diluted with H_2_O (400 mL) and extracted with ethyl acetate (600 mL, 300 mL × 2). The combined organic layers were washed with saturated aqueous NaCl (300 mL, 150 mL × 2), dried over Na_2_SO_4_, filtered, and concentrated under reduced pressure to yield a residue. The residue was purified by column chromatography (SiO_2_, Petroleum ether/Ethyl acetate = 10:1 to 2:1). Compound 3 (15.4 g, 34.2 mmol, 54.1% yield) was obtained as a yellow solid and confirmed by ^1^HNMR. Compound 3: ^1^HNMR (400 MHz, CDCl_3_) δ ppm 1.12 (d, J=7.2 Hz, 18 H) 1.27 - 1.33 (m, 3 H) 7.2 - 7.3 (m, 2 H) 7.4 (d, J=7.6 Hz, 1 H) 7.55 - 7.61 (m, 1 H) 7.66 - 7.73 (m, 2 H) 7.7 - 7.8 (m, 2 H) 7.9 (s, 1 H) 8.1 (d, J=7.6 Hz, 1 H).

Compound 4: To a solution of compound 3 (11 g, 24.5 mmol, 1 eq) in acetic acid (AcOH, 110 mL), palladium on carbon (Pd/C, 1.1 g, 1.7 mmol, 10% purity) was added under a nitrogen atmosphere. The suspension was degassed under vacuum and purged with hydrogen several times. The mixture was stirred under hydrogen (40-50 psi) at 60-70 ℃ for 24 hours. The mixture was stirred under hydrogen (40-50 psi) at 60-70 ℃ for an additional 6 hours. The reaction mixture was then cooled to 15-20 ℃ and filtered to remove the Pd/C. The pH of the filtrate was adjusted to 2-3 using 2 M HCl and extracted with ethyl acetate (EA, 200 mL, 100 mL × 2). The combined organic layers were washed with saturated aqueous NaCl (200 mL), dried over Na_2_SO_4_, filtered, and concentrated under reduced pressure to yield a residue. The residue was purified by column chromatography (SiO_2_, Petroleum ether/Ethyl acetate = 10:1 to 2:1). Compound 4 (7.9 g, 18.2 mmol, 74.1% yield) was obtained as a yellow oil and confirmed by ^1^HNMR. Compound 4: ^1^HNMR (400 MHz, CDCl_3_) δ ppm 1.16 (d, J=7.2 Hz, 18 H) 1.3 - 1.4 (m, 3 H) 4.6 (s 2 H) 7.1(dd, J=9.2 Hz, 2.4 Hz 1 H) 7.17 - 7.23 (m, 2 H) 7.3 (d, J=7.6 Hz, 1 H) 7.33 - 7.40 (m, 1 H) 7.48 - 7. 53 (m, 2 H) 7.7 (d, J=8.8 Hz 2 H) 8.1 (dd, J=7.6 Hz, 0.8 Hz 1 H).

Compound Target A: To a solution of compound 4 (7 g, 16.1 mmol, 1 eq) in THF (80 mL) was added TBAF (1 M, 20.9 mL, 1.3 eq) and the mixture was stirred at 15-20 ℃ for 1 hour. The reaction mixture was concentrated under reduced pressure to remove THF. The residue was adjusted to pH = 10-11 with 2 M NaOH and extracted with MTBE (2 × 50 mL). The aqueous layer was adjusted to pH = 2-3 using 2 M HCl and then extracted with EA (2 × 50 mL). The combined organic layers were washed with saturated aqueous NaCl (2 × 50 mL), then concentrated under reduced pressure to yield a residue. The residue was triturated with H_2_O (14 mL) at 15-20 ℃ for 20 minutes, then filtered and concentrated under reduced pressure to give compound Target A. Compound Target A (4 g, 13.7 mmol, 85.3% yield, 96.6% purity) was obtained as an off-white solid and confirmed by ^1^HNMR, LC-MS and with 96.6% purity by HPLC under 220 nm UV. Compound Target A: ^1^HNMR (400 MHz, DMSO-d6) δ ppm 4.5 (s, 2 H) 6.96 - 7.03 (m, 2 H) 7.1 (dd, J=8.4, 1.6 Hz, 1 H) 7.28 - 7.35 (m, 2 H) 7.39 (s, 1 H) 7.44 - 7.51 (m, 1 H) 7.65 (dd, J=11.6, 8.40 Hz, 2 H) 7.8 (dd, J=8.4, 0.8 Hz, 1 H) 9.7 (br s, 1 H) 12.9 (br s, 1 H)

***Synthesis of compound TB***

Compound 6: To a solution of compound 5 (10 g, 43.7 mmol, 1 eq) in 1,4-dioxane (100 mL) was added KOAc (8.6 g, 87.3 mmol, 2 eq), bis(pinacolato)diboron (11.1 g, 43.7 mmol, 1 eq), and Pd(dppf)Cl_2_ (1.3 g, 1.8 mmol, 0.04 eq) under a nitrogen atmosphere. The reaction mixture was stirred at 80-85 ℃ for 4 hours. The reaction mixture was diluted with H_2_O (60 mL) and extracted with MTBE (2 × 30 mL). The combined organic layers were dried over Na_2_SO_4_, filtered, and concentrated under reduced pressure to yield a residue. The residue was purified by column chromatography (SiO_2_, Petroleum ether/Ethyl acetate = 100/0 to 100/1). Compound 6 (10 g, 36.2 mmol, 83% yield) was obtained as a white solid and confirmed by ^1^HNMR. Compound 6: ^1^HNMR (400 MHz, CDCl_3_) δ ppm 1.2 (s, 12 H) 2.3 (s, 2 H) 3.9 (s, 3 H) 7.27-7.33 (m, 1H) 7.38 (d, J=7.6, 1H) 7.8 (d, J=7.6, 1H) 7.86 (s, 1H)

Compound 7: To a solution of compound 2 (8 g, 21.1 mmol, 1 eq) in 1,4-dioxane (80 mL) and H_2_O (19 mL) was added compound 6 (8 g, 28.9 mmol, 1.4 eq), Pd(dppf)Cl_2_ (1.5 g, 2.1 mmol, 0.1 eq), and K_2_CO_3_ (14.6 g, 105.4 mmol, 5 eq) under a nitrogen atmosphere. The reaction mixture was stirred at 80-85 ℃ for 8 hours. The mixture was then diluted with H_2_O (80 mL) and extracted with EA (2 × 40 mL). The combined organic layers were dried over Na_2_SO_4_, filtered, and concentrated under reduced pressure to yield a residue. The residue was purified by column chromatography (SiO_2_, Petroleum ether/Ethyl acetate = 100/0 to 100/1). Compound 7 (7.3 g, 16.3 mmol, 77.1% yield) was obtained as a yellow oil and confirmed by ^1^HNMR. Compound 7: ^1^HNMR (400 MHz, CDCl_3_) δ ppm 1.15 (d, J=7.6 Hz, 18 H) 1.29 - 1.37 (m, 3 H) 3.91 (s, 3 H) 4.15 - 4.18 (m, 2 H) 7.10 (dd, J=8.8, 2.4 Hz, 1 H) 7.13 - 7.18 (m, 2 H) 7.35 - 7.40 (m, 1 H) 7.42 - 7.47 (m, 1 H) 7.49 (s, 1 H) 7.69 (dd, J=8.4, 2.4 Hz, 2 H) 7.92 (br d, J=7.60 Hz, 1 H) 7.98 (s, 1 H)

Compound 8: To a solution of compound 7 (8.2 g, 18.3 mmol, 1 eq) in THF (80 mL) was added TBAF (1 M, 27.4 mL, 1.5 eq) and the mixture was stirred at 15-20 ℃ for 2 hours. The reaction mixture was diluted with H_2_O (80 mL) and extracted with MTBE (2 × 80 mL). The organic layer was washed with saturated aqueous NaCl (3 × 40 mL), dried over Na_2_SO_4_, filtered, and concentrated under reduced pressure to yield a residue. Compound 8 (8.4 g, crude) was obtained as a yellow gum and confirmed by ^1^HNMR. Compound 8: ^1^HNMR (400 MHz, CDCl_3_) δ ppm 3.89 - 3.94 (m, 4 H) 4.13 (s, 2 H) 7.06 - 7.12 (m, 2 H) 7.13 (dd, J=8.4, 1.2 Hz, 1 H) 7.34-7.38 (m, 1 H) 7.4 - 7.46 (m, 2 H) 7.66-7.70 (m, 2 H) 7.91 (d, J=7.6 Hz, 1 H) 7.96 (s, 1 H)

Compound Target B: To a solution of compound 8 (8 g, 27.4 mmol, 1 eq) in MeOH (80 mL) and H_2_O (160 mL) was added NaOH (2.7 g, 68.5 mmol, 2.5 eq). The mixture was stirred at 15-20 ℃ for 3 hours. The reaction mixture was concentrated under reduced pressure to remove MeOH. The residue was extracted with MTBE (2 × 50 mL). The combined aqueous layers were adjusted to pH = 2-3 and then extracted with EA (2 × 50 mL). The combined organic layers were washed with saturated aqueous NaCl (2 × 50 mL) and concentrated under reduced pressure to yield a residue with 97.5% purity by HPLC at 220 nm UV without further purification. Compound Target B (4 g, 14 mmol, 51.2% yield, 97.5% purity) was obtained as an off-white solid, confirmed by ^1^HNMR, LC-MS and with 97.5% purity by HPLC under 220 nm UV. Compound Target B: ^1^HNMR (400 MHz, DMSO-d6) δ ppm 4.11 (s, 2 H) 7.02 (dd, J=8.4, 2.4 Hz, 1 H) 7.06 (d, J=2.4 Hz, 1 H) 7.11 (dd, J=8.4, 1.2 Hz, 1 H) 7.39 - 7.45 (m, 1 H) 7.51 - 7.57 (m, 2 H) 7.68 (dd, J=8.8, 4.4 Hz, 2 H) 7.77 (br d, J=7.6 Hz, 1 H) 7.82 (s, 1 H) 9.72 (br s, 1 H) 12.8 (br m, 1 H).


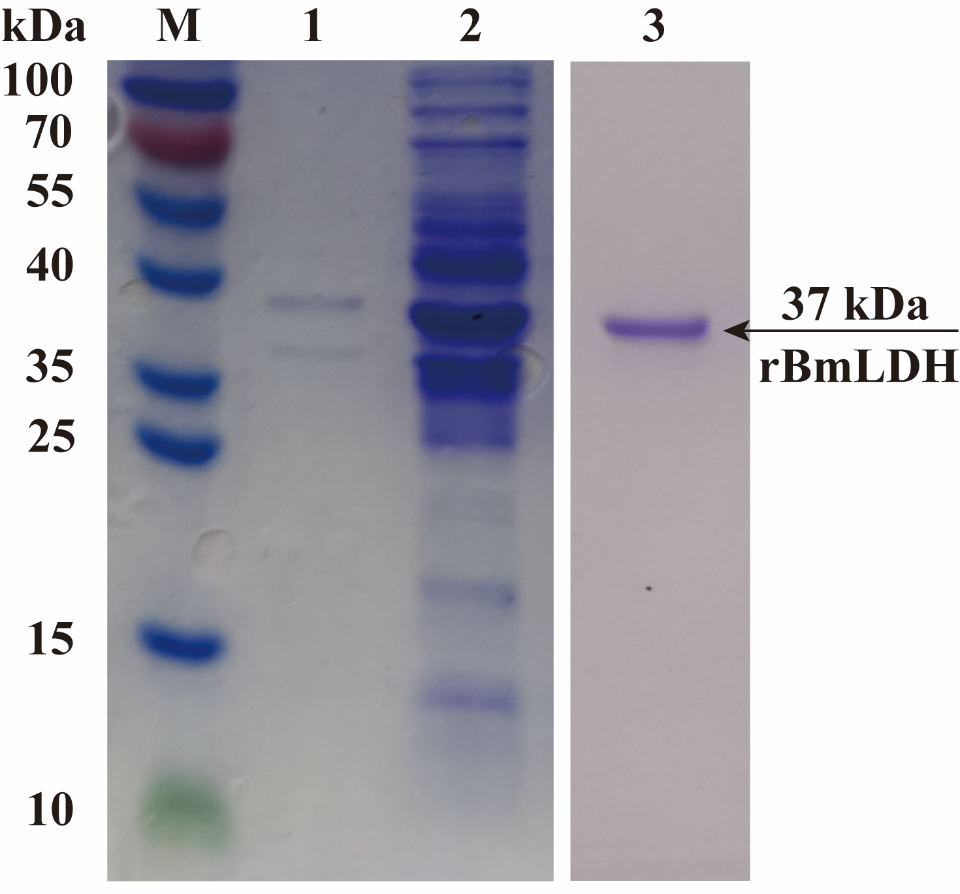


Fig. S2 SDS-PAGE of prokaryotic expression stained by Coomassie blue. Lane1 is the inclusion body. Lane2 is the supernatant (rBmLDH). Lane3 is the purified *Babesia micoti* lactate dehydrogenase protein. They were indicated by SDS-PAGE analysis.

Table S1. The docking score of five compounds.

| Compound ID | Structure | CDOCKER energy |
| --- | --- | --- |
| TA | 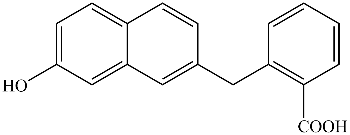 | -36 |
| TB | 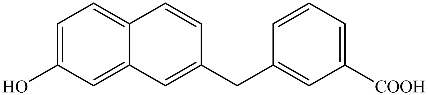 | -43.8 |
| Compound C | 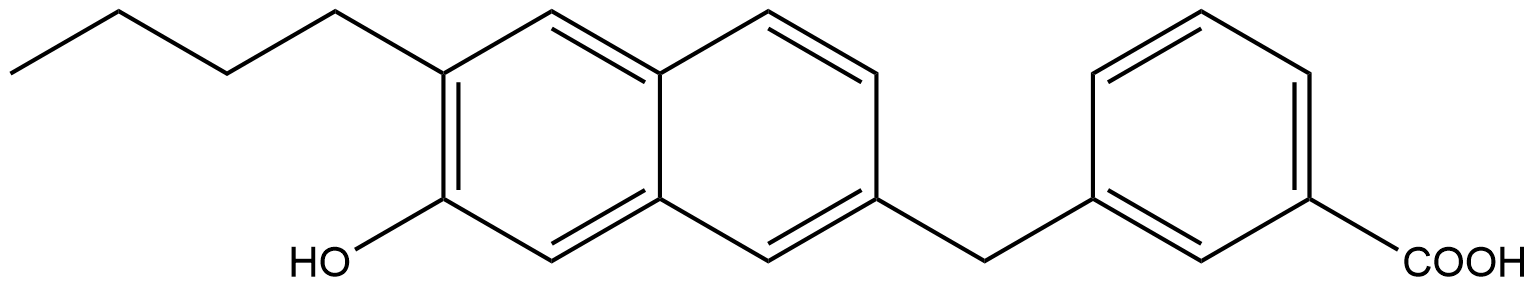 | 35.5 |
| Compound D | 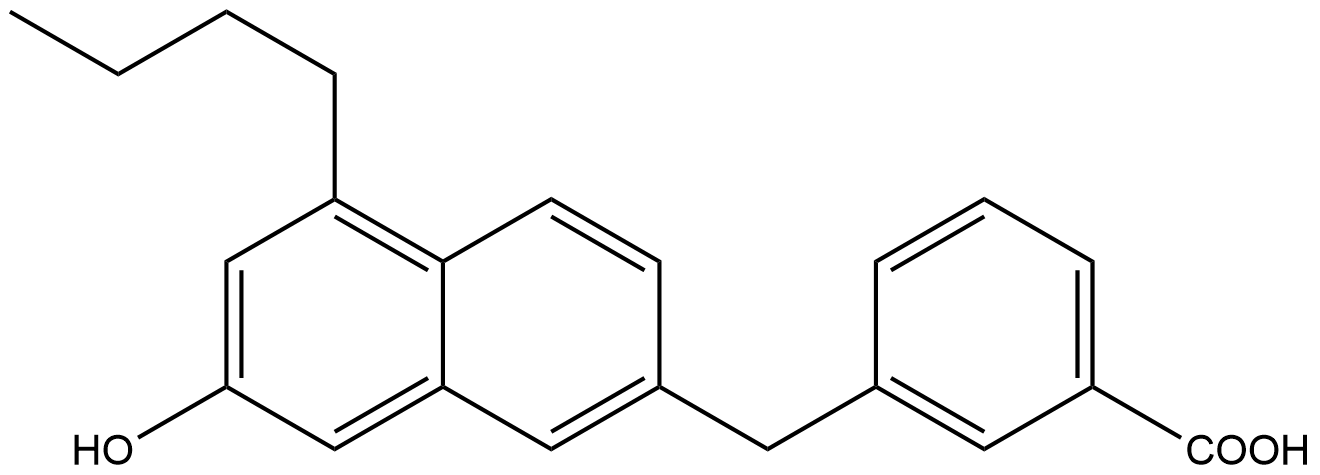 | 34.5 |
| Compound E | 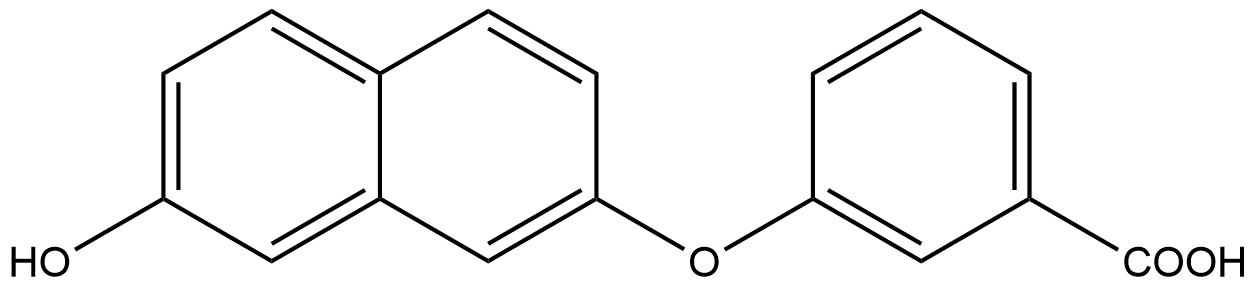 | 33.5 |
